# Double Consciousness: Explaining Racial Paradox in Later Life Psychological Well-Being

**DOI:** 10.1093/geroni/igaa057.2257

**Published:** 2020-12-16

**Authors:** Timothy Goler, Tirth Bhatta

**Affiliations:** 1 Norfolk State University, Newport News, Virginia, United States; 2 University of Las Vegas Nevada, Las Vegas, Nevada, United States

## Abstract

A substantial number of studies have documented paradoxical findings when examining race differences in later life psychological well-being. Despite experiencing significant structural disadvantages, Black older adults have been found to report significantly higher overall life satisfaction and lower depressive symptoms than White adults. This study relies on double consciousness framework which allows us to understand why satisfaction with material conditions (e.g., domain-specific life satisfaction) among Black older adults could differ from their evaluation of overall well-being (e.g., overall life satisfaction). Based on a survey of successful aging (n=409 aged 60 years or older) conducted by the Elderly Care Research Center (ECRC) in Cleveland, Ohio, we examined race differences in coping resources, and their role in shaping overall life satisfaction, domain-specific life satisfaction, and depressive symptoms. Findings show that Blacks on average have a higher likelihood of experiencing recent negative life events than their White counterparts. Despite adverse life circumstances, Blacks older adults expressed significantly higher overall life satisfaction than Whites. They, however, reported significantly lower domain-specific life satisfaction than their White counterparts. The differences in depressive symptoms between Black and White older adults was not statistically significant. The race differences in overall life satisfaction was explained by religiosity, religious coping, and social support. Education, income, and adverse life events were found to contribute to such differences in domain-specific life satisfaction. Our findings underscore the need to consider the unique role of racialized life course circumstances and coping resources in shaping disparities in later life psychological well-being.

